# Supplementary material for: Isolation and optimization of extracellular PHB depolymerase producer Aeromonas caviae Kuk1-(34) for sustainable solid waste management of biodegradable polymers
Source: PLoS One. 2022 Apr 14;17(4):e0264207. doi: 10.1371/journal.pone.0264207 (PMC9009665; doi:10.1371/journal.pone.0264207)
Supplement: Attachment — Submitted filename: PONE-D-21-27980.pdf [file pone.0264207.s003.pdf]

PLOS ONE

Isolation and optimization of extracellular PHB depolymerase producer Aeromonas caviae Kuk1-(34) for sustainable solid waste management of biodegradable polymers

--Manuscript Draft--

**Manuscript Number:**	PONE-D-21-27980
**Article Type:**	Research Article
**Full Title:**	Isolation and optimization of extracellular PHB depolymerase producer Aeromonas caviae Kuk1-(34) for sustainable solid waste management of biodegradable polymers
**Short Title:**	Novel extracellular PHB depolymerase producer Aeromonas caviae Kuk1-(34)
**Corresponding Author:**	Roohi NA<br>Integral University<br>Lucknow, UP INDIA
**Keywords:**	PHB; PHB depolymerase; Aeromonas caviae kuk1-(34); Soil burial method; Solid waste management
**Abstract:**	Bioplastics, synthesized by several microbes, accumulates inside the cells under stress conditions as a storage material. Microbial enzymes play a crucial role in their degradation. This research was carried to test the biodegradability of poly-β-hydroxybutyrate (PHB) utilizing PHB depolymerase enzyme, produced by bacteria isolated from sewage waste soil samples. Potent PHB degrader was screened based on zone of hydrolysis followed by PHB depolymerase enzyme activity. Soil burial method was employed to check their degradation ability at different incubation period of 15, 30 and 45 days at 37±2°C, pH 7.0 at 60% moisture with 1% microbial inoculum of Aeromonas caviae Kuk1-(34) (MN414252). After 45 days, 85.76% of the total weight of the PHB film was degraded. This degradation was confirmed with FTIR and SEM analysis, where presence of bacterial colonies on the surface of the degraded film, along with crest, holes, surface erosion and roughness are clearly visible. Media optimization was carried out in statistical mode using Plackett Burman (PB) and Central Composite Design (CCD) of Response Surface Methodology (RSM) by considering 10 factors. ANOVA, Pareto chart, response surface plots and F-value of 3.82 implies that the above statistical model was significant. Best production of PHB depolymerase enzyme (14.98 U/mL) was observed when Aeromonas caviae Kuk1-(34) was grown in a media containing 0.1% PHB, K_2HPO_4 (1.6 gm/L) at 27 °C for 7 days. This strain was found to be a suitable candidate to deal with solid waste management, where 94.4% of the total weight of the biodegradable PHB film was degraded after 45 days of incubation in soil burial method.
**Order of Authors:**	Mohammad Amir
	Naushin Bano
	Abu Baker
	Qamar Zia
	Saeed Banawas
	Mohd. Rehan Zaheer
	Mohammad Shariq
	Md Sarfaraz Nawaz
	Z. R. Azaz Ahmad Azad
	Roohi NA
**Additional Information:**	
**Question**	**Response**
**Financial Disclosure**	Dr Roohi is grateful to the Department of Science and Technology, India (DST-SERB) for providing financial support under the scheme of Early Career Research Award

Powered by Editorial Manager® and ProduXion Manager® from Aries Systems Corporation

Enter a financial disclosure statement that describes the sources of funding for the work included in this submission. Review the submission guidelines for detailed requirements. View published research articles from *PLOS ONE* for specific examples.<br><br>This statement is required for submission and **will appear in the published article** if the submission is accepted. Please make sure it is accurate.<br><br>**Unfunded studies**<br>Enter: *The author(s) received no specific funding for this work.*<br><br>**Funded studies**<br>Enter a statement with the following details:<br>• Initials of the authors who received each award<br>• Grant numbers awarded to each author<br>• The full name of each funder<br>• URL of each funder website<br>• Did the sponsors or funders play any role in the study design, data collection and analysis, decision to publish, or preparation of the manuscript?<br>• **NO** - Include this sentence at the end of your statement: *The funders had no role in study design, data collection and analysis, decision to publish, or preparation of the manuscript.*<br>• **YES** - Specify the role(s) played.<br><br>* typeset	(Project No. ECR/2017/001001). The funders had no role in study design, data collection and analysis, decision to publish, or preparation of the manuscript.
**Competing Interests**<br><br>Use the instructions below to enter a competing interest statement for this submission. On behalf of all authors, disclose any competing interests that could be perceived to bias this work—acknowledging all financial support and any other relevant financial or non-financial competing interests.<br><br>This statement is required for submission and **will appear in the published article** if the submission is accepted. Please make	The authors have declared that no competing interests exist.

sure it is accurate and that any funding sources listed in your Funding Information later in the submission form are also declared in your Financial Disclosure statement.<br><br>View published research articles from *PLOS ONE* for specific examples.<br><br>**NO authors have competing interests**<br><br>Enter: *The authors have declared that no competing interests exist.*<br><br>**Authors with competing interests**<br><br>Enter competing interest details beginning with this statement:<br><br>*I have read the journal's policy and the authors of this manuscript have the following competing interests: [insert competing interests here]*<br><br>* typeset	
**Ethics Statement**<br><br>Enter an ethics statement for this submission. This statement is required if the study involved:<br><br>• Human participants<br>• Human specimens or tissue<br>• Vertebrate animals or cephalopods<br>• Vertebrate embryos or tissues<br>• Field research<br><br>Write "N/A" if the submission does not require an ethics statement.<br><br>General guidance is provided below. Consult the submission guidelines for detailed instructions. **Make sure that all information entered here is included in the Methods section of the manuscript.**	N/A

Powered by Editorial Manager® and ProduXion Manager® from Aries Systems Corporation

**Format for specific study types**<br><br>**Human Subject Research (involving human participants and/or tissue)**<br>• Give the name of the institutional review board or ethics committee that approved the study<br>• Include the approval number and/or a statement indicating approval of this research<br>• Indicate the form of consent obtained (written/oral) or the reason that consent was not obtained (e.g. the data were analyzed anonymously)<br><br>**Animal Research (involving vertebrate animals, embryos or tissues)**<br>• Provide the name of the Institutional Animal Care and Use Committee (IACUC) or other relevant ethics board that reviewed the study protocol, and indicate whether they approved this research or granted a formal waiver of ethical approval<br>• Include an approval number if one was obtained<br>• If the study involved *non-human primates*, add *additional details* about animal welfare and steps taken to ameliorate suffering<br>• If anesthesia, euthanasia, or any kind of animal sacrifice is part of the study, include briefly which substances and/or methods were applied<br><br>**Field Research**<br><br>Include the following details if this study involves the collection of plant, animal, or other materials from a natural setting:<br>• Field permit number<br>• Name of the institution or relevant body that granted permission	
**Data Availability**<br><br>Authors are required to make all data underlying the findings described fully available, without restriction, and from the time of publication. PLOS allows rare exceptions to address legal and ethical concerns. See the PLOS Data Policy and FAQ for detailed information.	Yes - all data are fully available without restriction

A Data Availability Statement describing where the data can be found is required at submission. Your answers to this question constitute the Data Availability Statement and **will be published in the article**, if accepted.<br><br>**Important:** Stating ‘data available on request from the author’ is not sufficient. If your data are only available upon request, select ‘No’ for the first question and explain your exceptional situation in the text box.<br><br>Do the authors confirm that all data underlying the findings described in their manuscript are fully available without restriction?	
**Describe where the data may be found in full sentences. If you are copying our sample text, replace any instances of XXX with the appropriate details.**<br><br>• If the data are **held or will be held in a public repository**, include URLs, accession numbers or DOIs. If this information will only be available after acceptance, indicate this by ticking the box below. For example: *All XXX files are available from the XXX database (accession number(s) XXX, XXX.).*<br>• If the data are all contained **within the manuscript and/or Supporting Information files**, enter the following: *All relevant data are within the manuscript and its Supporting Information files.*<br>• If neither of these applies but you are able to provide **details of access elsewhere**, with or without limitations, please do so. For example:<br><br>*Data cannot be shared publicly because of [XXX]. Data are available from the XXX Institutional Data Access / Ethics Committee (contact via XXX) for researchers who meet the criteria for access to confidential data.*<br><br>*The data underlying the results presented in the study are available from (include the name of the third party*	N/A

and contact information or URL).<br>• This text is appropriate if the data are owned by a third party and authors do not have permission to share the data.<br><br>* typeset	
Additional data availability information:	

Manuscript

Click here to access/download;Manuscript;MS-Aeromonascviae (20.08.21).doc

Isolation and optimization of extracellular PHB depolymerase producer *Aeromonas caviae* Kuk1-(34) for sustainable solid waste management of biodegradable polymers

Mohammad Amir[1], Naushin Bano[1], Abu Baker[1], Qamar Zia[2, 3], Saeed Banawas[2, 3], Mohd. Rehan Zaheer[4], Mohammad Shariq[4], Md Sarfaraz Nawaz[5], Mohd. Farhan Khan[6, 7], Z. R. Azaz Ahmad Azad[8], Roohi[1, *]

[1]Protein Research Laboratory, Department of Bioengineering, Integral University, Lucknow, India
[2]Health and Basic Science Research Centre, Majmaah University, Majmaah, Saudi Arabia
[3]Department of Medical Laboratory Sciences, College of Applied Medical Sciences, Majmaah University, Majmaah, Saudi Arabia
[4]Department of Physics, Faculty of Science, Jazan University, Jazan, Saudi Arabia
[5]Department of Chemistry, Faculty of Science, Jazan University, Jazan, Saudi Arabia [6]Department of Science, Gagan College of Management and Technology, Aligarh, India
[7]Nano Solver Lab, Department of Mechanical Engineering, Z. H. College of Engineering & Technology, Aligarh Muslim University, Aligarh, India
[8]Department of Post-Harvest Engineering and Technology, Aligarh Muslim University, Aligarh, India

Short Running Title: Extracellular PHB depolymerase from *A. caviae* Kuk1-(34)

***Corresponding author**:

Dr. Roohi
E-mail: roohi0607@gmail.com (R)

Abstract

Bioplastics, synthesized by several microbes, accumulates inside the cells under stress conditions as a storage material. Microbial enzymes play a crucial role in their degradation. This research was carried to test the biodegradability of poly-β-hydroxybutyrate (PHB) utilizing PHB depolymerase enzyme, produced by bacteria isolated from sewage waste soil samples. Potent PHB degrader was screened based on zone of hydrolysis followed by PHB depolymerase enzyme activity. Soil burial method was employed to check their degradation ability at different incubation period of 15, 30 and 45 days at 37±2°C, pH 7.0 at 60% moisture with 1% microbial inoculum of *Aeromonas caviae* Kuk1-(34) (MN414252). After 45 days, 85.76% of the total weight of the PHB film was degraded. This degradation was confirmed with FTIR and SEM analysis, where presence of bacterial colonies on the surface of the degraded film, along with crest, holes, surface erosion and roughness are clearly visible. Media optimization was carried out in statistical mode using Plackett Burman (PB) and Central Composite Design (CCD) of Response Surface Methodology (RSM) by considering 10 factors. ANOVA, Pareto chart, response surface plots and F-value of 3.82 implies that the above statistical model was significant. Best production of PHB depolymerase enzyme (14.98 U/mL) was observed when *Aeromonas caviae* Kuk1-(34) was grown in a media containing 0.1% PHB, K_2HPO_4 (1.6 gm/L) at 27 ℃ for 7 days. This strain was found to be a suitable candidate to deal with solid waste management, where 94.4% of the total weight of the biodegradable PHB film was degraded after 45 days of incubation in soil burial method.

Keywords: PHB; PHB depolymerase; *Aeromonas caviae* kuk1-(34); Soil burial method; Solid waste management

1. Introduction

Plastics play an essential role in daily life; hence, its requirement increased tremendously from 1.5 million tonnes in 1950 (Chidambarampadmavathy et al. 2017) to approximately 300 million tons in 2015 (Sharma and Jain 2020). These synthetic polymers are usually low-cost, but have a major negative impact on our environment (Getachew and Woldesenbet 2016). Due to its non-degradable nature, plastics are the main culprit of environmental nuisance. Despite many recycling efforts, the results of plastics disposal in municipal landfills still creates major problems. Consequently, many attempts have been undertaken to generate renewable, degradable, and recyclable materials, i.e., green materials for sustainability (Wu 2012; Agustin et al. 2014). Poly(lactic acid) (PLA), poly(butylene succinate adipate) (PBSA), polycaprolactone (PCL), and poly(hydroxyalkanoates) (PHAs) are few biodegradable aliphatic polymers, which are able to replace the polyethylene (PE) and polystyrene (PS) that took hundreds or thousands of years to degrade (Wu, 2012).

PHAs are produced by the diversity of microorganisms as carbon and energy storage material under stress conditions. Poly(hydroxybutyrate) (PHB) is the most commonly occurring PHA, which comprises of packed monomers of ®-3-hydroxybutyrate (R3HB) (Hisano et al. 2006; McGenityet al. 2010). PHB depolymerase (EC 3.1.1.75) is an extracellular and intracellular hydrolyzing enzymes that degrades PHB effectively. Partially structured (denatured) PHB is degraded by extracellular depolymerase while intracellular depolymerase operate on unstructured (native) PHB. A plethora of reports are available on the biodegradation of PHAs in the terrestrial, marine, soil environment. As a result, several microbes has been characterised that are responsible for the degradation of these polymers (Shah et al. 2007). In the present study, the biodegradation of the PHB polymer from the novel bacterial enzyme, isolated from the soil waste, was observed. Their detailed statistical production optimizations were also studied including the soil burial applications for sustainability of the environment.

2. Materials and Methods

2.1. Polymer studied

Poly[(*R*)-3-hydroxybutyric acid] (PHB) powder with linear formula of $[COCH_2CH(CH_3)O]_n$ was purchased from Sigma-Aldrich, CAS Number: 29435-48-1.

2.2. Sample collection and isolation of PHB degrading microbes

Soil samples from sewage sludge in semi-solid form were taken from four different sources of Lucknow, Uttar Pradesh, India for the isolation of potent PHB depolymerase producing microorganisms. Samples were processed by serial dilution following spread plate method on Bushnell Hass medium and incubated for 48 hours at 37 ºC for the proliferation of microbial growth and isolation of microorganisms (Mikkili et al. 2014).

2.3. Screening of PHB depolymerase producing isolates via zone of hydrolysis

PHB depolymerase producer were screen out by clear zone assay on Bushnell Hass Medium (g/L): Magnesium sulphate, 0.20 g; Calcium chloride anhydrous, 0.02 g; Potassium di-hydrogen phosphate, 1.0 g; Di-potassium hydrogen phosphate, 1.0 g; Ammonium nitrate, 1.0 g; Ferric chloride, 0.05 g with 0.15% PHB powder sonicated in an ultrasonic water bath (Labman-LMUC 3, 40KHz and 100W) at 40 ºC for 20 min followed by the addition of 2% agar powder to generate solid media. After the sonication, plates were incubated for 7 days at 37 ºC and then the clear hydrolysis zone was measured.

2.4. PHB depolymerase assay

PHB depolymerase assay was performed as per the modified method of Kobayashi et al. (1999). 50 mM Tris-HCL buffer (pH 7.0) with 0.15% of PHB powder was suspended and subjected to sonication immersed in ultrasonic water bath (40 KHz and 100 W) for 30 min. In 0.9 mL of the substrate suspension, 0.1 mL of the culture supernatant was added and incubated for 25 min at 37 ºC. Activity of the decrease in the turbidity of the PHB suspension was measured at OD_{650} against the substrate buffer blank (1 mL of Tris HCL buffer) (Shah et al. 2007).

2.5. Morphological, physiological, biochemical identification with 16S rRNA gene sequencing analysis

The positive bacterial isolates that gave clear zone of hydrolysis on PHB plates were identified in accordance with Bergey's Manual of Determinative Bacteriology. Detailed analysis was done for morphological, physiological, and biochemical identification (Table 3 (B)).The 16S rRNA gene sequence analysis was performed by Biokart, Bangalore, India Pvt. Ltd. using forward primer sequence 27F and backward primer sequence 149R (Bano et al. 2019). DNA baser was used to evaluate sequences and comparison of nuclear sequences further analysed accessible at the National Center for Biotechnology Information (NCBI)

using BLAST (Basic Local Alignment Search Tool) search tool. The phylogenetic tree, as implemented in MEGA X from the nucleotide sequences, has been developed using the neighbouring ClustalW method (Satti et al. 2020).

2.6. Preparation of PHB film

PHB film was prepared by solvent casting method where 0.1 g of PHB powder (Sigma-Aldrich) was suspended in 30 mL of chloroform, while kept on magnetic stirrer for 20 min at 45 ºC. PHB-suspension was poured into the clean autoclaved glass petri plates and chloroform was then vaporized. Petri plates were further incubated at 37 ºC for 24 h, resulting in the formation of PHB films about 10 cm in diameter and 2 mm thickness (Gouda et al.2012).

2.7. Soil burial biodegradation analysis with PHB film

The weight in the change of PHB polymer film was calculated before and after the treatment with the positive bacterial strains. PHB films (~0.0245 g)were buried in autoclaved soil in pots (at 3 cm depth) at the ambient temperature (35–37 °C) with the moisture content uphold to 60% and presence of mineral salts and incubated for different time period (15, 30 and 45 days). The pre-weighed PHB polymer films were then taken off from the soil after different incubation time and washed several times by distilled water to remove the soil particles and then dried at room temperature (Bano et al. 2019). The polymer degradation was calculated by the given formula;

$$Degradation\,\% = \left[\frac{(Wif - Wff)}{Wif}\right] 100$$

(*Wif*= initial weight of films, *Wff* = final weight of buried films)

Soil fragmented PHB film surface was analysed using Scanning electron microscopy (SEM) and FTIR for detailed visualization (Bano et al. 2019). Before SEM analysis, the PHB films were fixed in 4% (v/v) glutaraldehyde in sodium cacodylate buffer (100 mM pH 7.2). After the glutaraldehyde fixation, the PHB films were again fixed in 20 g/L of aqueous osmium tetroxide and after drying to the critical point, examined under SEM (Fig. 5) (Altaee et al. 2016).

2.8. Fourier-transform infra-red (FTIR) spectroscopic analysis

FTIR spectra of PHB polymer film were obtained using a Perkin Elmer System 2000 Fourier transform infrared spectrometer. A small amount of the PHB polymer film was immersed in organic solvent, milled with KBr and pressed into a transparent film for FTIR analysis. FTIR spectra were collected over the range of 4000 to 450 cm^{-1} (Ni and Wang, 2002). FTIR determined functional groups in the degradation patterns were compared with the control (Sayyed et al. 2020).

2.9. Statistical optimization of the media variables for PHB depolymerase enzyme production using Placket-Burman (PB) experimental design

PB experimental design was used in the initial phase of optimization using Minitab 19. In this experiment, ten independent variables were used and each variable was checked at a high (+1) and a low (-1) values, as summarised in Table 1 (A). All the experiments were carried out in triplicates and repeated twice. PHB depolymerase activity was used as a response based on experimental design and polynomial model of the first order as follows:

$$Y=\beta_0\Sigma\beta\text{i}Xi$$

where Y is the response (enzyme production), β_0 is the model intercepts, βi is the linear coefficient and Xi is the level of the independent variable. This model was used to screen and assess the key factors influencing the response. The p-value ($\leq$0.05) was the probability that defines the magnitude of a contrast co-efficient arising from the variability of the random process and was calculated using variance analysis (ANOVA). Pareto chart analysis drawn with standardized effects and applying the F-value confirmed these significant values, the importance of each effect was observed (Wani et al. 2015).

2.10. Statistical optimization of the PB variables for PHB depolymerase enzyme production using Response surface methodology (RSM)

Response surface central composite design (CCD) specifies the optimal concentration of the significant variables and the interacting effect of media ingredient obtained in PB design. Five variables namely time, temperature, PHB, KH_2PO_4, and K_2HPO_4 with 32 runs of experiments were constructed which holds other variable constants. A high (+2) to low (-2) values have been checked for each variable, as summarised in Table 1 (B). The model was validated by analysis of variance (ANOVA) and response surface plots to ensure efficiency.

2.11. Application of isolated bacterial strainfor soil bioremediation by soil burial method in aspects of solid waste management

The biodegradation of the PHB based biofilm treated with the isolated bacterial strain by soil burial method in all the statistically optimized condition was carried out in aspects of solid waste management. About 200 g of autoclaved soil was taken in each soil pots and PHB based biofilm was treated with the isolated bacterial isolate, kept under it and were incubated for further 15, 30 and 45 days at optimized temperature and ambient moisture. The biodegradation of the PHB film was observed in terms of weight loss after 15, 30 and 45 days, compared with untreated control PHB-based biofilm and were expressed in the percentile reduction in the weight of PHB film (Sayyed et al. 2020).

3. Results

3.1. Soil sample collection

In the present study, total four soils samples were collected from different dumping zone sites of Lucknow, which gave maximum number of bacterial strains. These potent producers of PHB depolymerase enzymes were utilized for the degradation of PHB-based bioplastics. Total 22 positive PHB degraders were obtained (Table 2), based on their ability to form zone of hydrolysis on PHB agar plate (Figure 1A and 1B).

3.2. Screening of PHB depolymerase isolates based on zone of hydrolysis

Out of total 22 bacterial strains, three were selected on the basi[illegible]f zone diameter and were named as CB2-(20), Kuk1-(34)& CA6-(55) (Figure 1 C, 1D and 1E). The zone diameters were observed in the range of 2.5-11.3 mm (Table 3A), when incubated for 7 days at 37 °C.

3.3. PHB depolymerase activity of positive isolates

The degradation ability of 22 positive isolates were also checked by PHB depolymerase enzyme assay. The isolates, CB2-(20), Kuk1-(34) & CA6-(55), exhibited potent activity in the range of 1.55-2.06 U/mL/min (Table 3A).Among these three significant PHB degraders, Kuk1-(34) gave best PHB depolymerase activity (2.06 U/mL/min) and maximum zone of hydrolysis (11.3 mm) and, therefore, was selected for further study.

3.4. Morphological, biochemical, physiological and phylogenetic analysis

All the physical and physiological characteristics of the bacterial strain Kuk1-(34) is represented in Table 3B. Phylogenetic analysis of the Kuk1-(34) strain was concluded with the help of alignment and cladistic analysis of homologous sequence of the known bacteria. The 16S rRNA sequences was submitted in NCBI through GenBank and their identity was performed through BLAST. The phylogenetic tree of the submitted strain in the NCBI is shown in Figure 2 and the strain was identified as *Aeromonas caviae* Kuk1-(34) sp. with accession number MN414252 (Table 4).

3.5. Weight loss analysis of PHB film by soil burial method

Different weights of PHB films were buried in 200 g of sterile soil inside the pot with different strains separately. It was observed that the weight of PHB film was maximally reduced after the pre-treatment with strain *A. caviae* Kuk1-(34) sp. after 45 days as shown in Table 5 as compared to the control (Figure 3). This isolate showed significant tendency to degrade biodegradable plastics, since it uses the PHB as a sole carbon and energy source.

3.6. SEM Analysis of soil buried PHB films

In the present study, soil buried PHB films of 15, 30 and 45 days were analysed with the help of SEM. It was clearly observed that number of holes, crest, surface erosion, and roughness was very significant on all the PHB films as compared to the control. Several bacterial colonies of *A. caviae* Kuk1-(34) sp. were seen attached on the surface of the PHB films, which implies that this species actually secretes extracellular depolymerase enzyme that degrades the PHB film (Figure 4).

3.7. FTIR Analysis of soil buried PHB films

FTIR analysis of soil buried PHB film treated with *A. caviae* Kuk1-(34) sp. showed changes in the functional groups and significant shift of wave-numbers, indicating the biodegradation of PHB film. It was observed that the control FTIR chromatogram peak for functional group O-H appearing at 3435.25 cm^{-1} gets shifted to 3392.51 cm^{-1} after 15 days incubation with *A. caviae* Kuk1-(34) sp. This peak further changed to 3401.97 cm^{-1} and 3409.23 cm^{-1} with intensity of 62.2 and 44.67, when incubation time was increased to 30 and 45 days respectively. The next functional group ester bond present in the control showed peak at 1729.63 cm^{-1} with intensity 32.46; while in *A. caviae* treated samples after 15, 30 and 45 days shows the peak shifted at 1623.16 cm^{-1} with intensity of 49.21, 1724.7 cm^{-1} with intensity 60.4 and 1633.04 cm^{-1} with intensity 45.92, respectively. There is the formation of new bond

and presence of new functional group after degradation at 45 days having peak at 2923.08 cm^{-1} with intensity of 47.84, identifiable as -C-H functional group. Another peak at 1047.18 cm^{-1} with intensity of 46.02 is indicative of -C-O functional group. These changes in wavelength frequency of major functional groups in degrading samples as compared to control give preliminary proof of biodegradation of PHB film by *A. cavia e*Kuk1-(34) sp. after incubation for 45 days (Figure 5).

3.8. Statistical optimization to determine the interactive impact of *Aeromonas caviae* Kuk1-(34) sp. on enzyme production using PlackettBurman design

For PlackettBurman analysis, ten independent variables were used and the experiment was carried out with 12 runs in different combinations to identify the significant parameters for extracellular PHB depolymerase production. Higher (+1) and lower (-1) values have been screened for each variable (Table 1 A). Experimental data was statistically analysed (Table 6A) using F-test for ANOVA. The model F-value of 202.74 implies that the model was significant and the *p*-values were used as a tool to check the significance of each parameter (Table 6B). Thus, *p*-values less than 0.05 denoted the significance of the factors on enzyme production. The Pareto Chart in Figure 6A illustrates the order of significance of the variables affecting the Kuk1-(34) production in Plackett-Burman design. Among the ten variables, temperature showed the highest positive effect followed by time, PHB and K_2HPO_4, while KH_2PO_4 comes near to the positive and effective parameter. The negative effect was shown by NH_4NO_3, pH, $CaCl_2$, $FeCl_3$ and $MgSO_4$. Therefore, for the next step of optimization, the optimum level of time, temperature, PHB, K_2HPO_4 and KH_2PO_4 were checked by the Response surface methodology using central composite design (CCD). The model equation for enzyme production (Y) can be written as:

$$Y = 4.1019 - 1.3123\ \text{Time} - 2.2919\ \text{Temperature} - 0.6121\ \text{pH} + 1.0083\ \text{PHB} - 0.1299\ MgSO_4 - 0.2604\ CaCl_2 - 0.9078\ KH_2PO_4 + 0.9406\ K_2HPO_4 - 0.7756\ NH_4NO_3 + 0.1359\ FeCl_3$$

3.9. Statistical optimization of significant factors of PB plots by Central Composite Design (CCD) for PHB depolymerase enzyme production

Significant factors of the PB experiment were then tested by the CCD (Table 7A) to evaluate the optimized value for extracellular PHB depolymerase production. Time, temperature, PHB, K_2HPO_4 and KH_2PO_4 were assessed using Minitab 19 software. Each variable was tested from high (+2) to low (–2) levels (Table 1 B). The ANOVA result showed that the

regression is statistically significant for enzyme production. The F-value of 3.82 implies that the model was significant (Table 7B). The Pareto Chart (Figure 6B) illustrates the order of significance of the variables affecting the production of enzyme from *Aeromonas caviae* Kuk1-(34) sp. The autonomous K_2HPO_4 showed the greatest beneficial impact among all the factors followed by autonomous time variable, autonomous KH_2PO_4 variable and finally interaction between time and PHB that also showed valuable impact. Each response surface for enzyme activity indicated a clear peak, which means that the optimum point was inside the design boundary level (Figure 6C). The effect of time and temperature on the enzyme activity while keeping PHB, K_2HPO_4 and KH_2PO_4 at zero level was depicted. The response surface plot showed that the maximum production of enzyme could be attained at optimum temperature (27ºC) and maximum incubation time (3 days). From the result, the enzyme production increased exponentially with decrease in the incubation time and keeping temperature at optimum level. By applying multiple regression analysis on the experimental data, the following second order polynomial equation was obtained to describe the enzyme production efficiency:

$$Y = 14.308 + 0.0044\ \text{Temperature} + 0.1495\ \text{Time} + 0.0225\ \text{PHB} + 0.1687\ K_2HPO_4 - 0.1437\ KH_2PO_4 + 0.0069\ \text{Temperature} * \text{Temperature} + 0.0119\ \text{Time} * \text{Time} - 0.0809\ \text{PHB} * \text{PHB} + 0.0681\ K_2HPO_4 * K_2HPO_4 - 0.0369\ KH_2PO_4 * KH_2PO_4 - 0.0822\ \text{Temperature} * \text{Time} + 0.0521\ \text{Temperature} * \text{PHB} - 0.0248\ \text{Temperature} * K_2HPO_4 - 0.0252\ \text{Temperature} * KH_2PO_4 - 0.1703\ \text{Time} * \text{PHB} + 0.0006\ \text{Time} * K_2HPO_4 + 0.0844\ \text{Time} * KH_2PO_4 - 0.0177\ \text{PHB} * K_2HPO_4 + 0.0002\ \text{PHB} * KH_2PO_4 + 0.0821\ K_2HPO_4 * KH_2PO_4$$

The contour plot (Figure 6D) is used to predict the optimal levels of component for different test variables. Two variables, time and temperature were used to observe the enzyme activity of the PHB depolymerase from *A. caviae* Kuk1-(34) sp. while keeping the hold values of PHB, KH_2PO_4 and K_2HPO_4 as zero. It is clearly observed from the contour plot that the PHB depolymerase from *A. caviae* Kuk1-(34) sp. gave maximum enzyme activity with the increase in the incubation time (9 days) and decrease in the temperature (17ºC).

4. Application of *A. caviae* Kuk1-(34) sp. for soil bioremediation by soil burial method in aspects of solid waste management

The optimized parameters obtained from the CCD were further used for the application in aspects of solid waste management by soil burial method. Autonomous factors like time, KH_2PO_4, K_2HPO_4 with the (+1) values and the combined interaction of time and PHB with (-1) values were used for the soil burial method treated with *A. caviae* Kuk1-(34) sp. For 15, 30 and 45 days as compared with the control (without treatment). The PHB polymer film of 8 cm in diameter having a pre-weight of 0.025 g was buried in soil pots containing 200 g of soil while the humidity was maintained to 60%. The whole setup was kept at ambient temperature (35-37°C) and amended with the mineral solutions to sustained the necessity of the mineral salts. After the incubation of 15, 30 and 45 days, soil buried PHB polymer film was taken from the soil pots (Figure 7). These PHB polymer film was cleaned gently with brush and washed with distilled water to remove the soil particles. The decrease in the weight of the PHB films are summarised in Table 8, when degraded by the treatment with *A. caviae* Kuk1-(34) sp. using optimized factors from CCD ([illegible]ure 7). The weight loss degradation of the PHB film after 45 days of incubation was 94.4 % after the soil burial method, which also indicates that the CCD model validate the optimized values that can be used on industrial level for the degradation of PHB based bioplastics.

5. Discussion

Rapid increase in the production and utilization of synthetic plastics on the daily basis and its non-degradability in nature leads to introduction of biodegradable PHB-based bioplastics. PHB is a biopolymer produced by many microorganisms in stress condition and accumulated as the intracellular storage material as a reserved carbon source (Wani et al. 2016). In the present study, soil samples were collected from the sewage waste from different sources of Lucknow. Out of total 22 positive isolates, 3 isolates showed significant growth and exhibited variable degree of PHB hydrolysis on BHM media, containing PHB as the single carbon source. The largest zone (11.3 mm) was produced by the bacterial isolate *A. caviae* Kuk1-(34) sp. on the BHM media containing 0.15% (w/v) of PHB when incubated for 7 days at 37 ºC. This isolate was consequently selected as the prominent PHB depolymerase producer for the degradation PHB based bioplastic.

In literature, several articles are available regarding the isolation of different bacterial and fungal species that are potent degrader of PHB-based bioplastics. Mergaert et al. (1993) isolated PHB and P(3HB-co-3HV) copolymer, Elbanna et al. (2004) described *Pseudomonas indica* K2 and *Schlegelella* thermodepolymerase as the PHA degrader, while Sayyed et al.

(2019) isolated PHB degrader strain from the soil microbes on MSM containing PHB as sole carbon source. Typical enzyme assays for PHB depolymerase has also been described earlier by many researchers. In our study, the isolated strain *A. caviae* Kuk1-(34) sp. was analysed for the PHB depolymerase assay. Previous research conducted by Sayyed et al. (2020) using *Stenotrophomonas* sp. RZS7 yielded 0.721 U/mL/min of PHB depolymerase after 4 days of incubation; however, in our case, the strain *A. caviae* Kuk1-(34) sp. yielded about 2.0623 U/mL/min of PHB depolymerase after the incubation for 7 days at 37 ºC (Sayyed et al. 2020). Similar enzyme activities for the PHB depolymerase have been reported by many researchers (Wang et al. 2002; Zhou et al. 2009). Almost 86% of its total weight of the PHB film was degraded after the pre-treatment with *A. caviae* Kuk1-(34) sp. after the incubation for 45 days in the soil, while no changes was observed in control PHB film (without pre-treatment). This data is quite significant than those of Banoet al. (2019) regarding the degradation of PHB film using *Paenibacillu salvei* PHB28.

SEM analysis of the PHB polymer film was used to observe the progress of degradation. In the present study, surface analysis of the treated PHB polymer film after the incubation of 15, 30 and 45 days in the soil burial method showed significant variation in the morphology of the PHB polymer film. There was clear visualization of cracks and holes on the surface of the PHB polymer film. Morphological analysis of polyethylene surface comparing with the control film was done by SEM and was reported by Gautam and Kaur (2013). Another study conducted by Calabia and Tokiwa (2009), observed the growth of *Streptomyces* sp. SC-17 on the surface of the PHB film which were responsible for the presence of crust and holes (Tokiwa et al. 2009; Sayyed et al. 2020). Microbial degradation from the soil burial method of the PHB film was also reported by Wen and Lu in 2012 (Wen and Lu 2012). A similar study conducted by Bano et al.(2019) reported 15% of the PHB film degradation in soil after 45 days of incubation and observed the surface morphology under SEM (Bano et al. 2019).

The treated PHB films were also analysed by FTIR. The findings suggest that the chemical structure of PHB possess molecules terminated by a hydroxyl and a carboxyl group. The hydroxyl and carboxyl end functional groups showed peaks at approximately 3435 cm^{-1} and 1729 cm-^{1}, respectively. Previous characteristics of the PHB vibrations was found to be around 1290 cm^{-1} and 980 cm^{-1}. The peak at 1290 cm^{-1} determine the –C-O-C- group, while the peak at 980 cm^{-1} can be allocated to bending vibrations of olefinic -C-H (Ong et al. 2011). As the deprivation begins with time, it incorporates vinyl (crotonate) ester and carboxyl groups end groups in PHB structure (Calil 2001). Consequently, a steady rise in crotonate

ester groups with extrusions paths can be expected, as well as a decline in hydroxyl groups available in the original polymer. The absorption band assign to stretching vibrations of double carbon/carbon bond, -C=C-, is to shown at around between 1600 and 1700 cm^{-1} (Vogel et al. 2007). The band at 1729 cm^{-1}, allocated originally to the carbonyl absorption band in the infrared spectra, is shifted to 1633 cm^{-1} when combined with vinyl end groups after 45 days (Yu et al. 2005). The availability of absorption bands associated to the formation of new chemical groups due to deprivation mechanisms of polymer was noticed at 2923.08 cm^{-1} and 1047.18 cm^{-1}, respectively.

To maximise enzyme output yield, the PB design and RSM were implemented, which was demonstrated as an effective screening method for significant medium components and their optimum amounts for full yield. In this study, to increase the enzyme production by *A. caviae* Kuk1-(34) sp., four factors were screened out from 10 factors by Placket Burman design. These 4 factors were further optimized for the RSM using CCD, where maximum enzyme activity of *A. caviae* Kuk1-(34) sp. was obtained at 14.98 U/min/mL ($p<0.05$). Shivkumar (2012) reported *Penicillium expansum* for the production of depolymerase enzyme using the Placket Burman design. Bansal et al.(2014) reported *Aeromonas punctata* sp. for the production and optimization of depolymerase enzyme using PB design and RSM (Bansal et al. 2014). Similarly, Tseng et al. (2007) studied the production of PHB depolymerase from *E. minima* W2 (PhaZEmi) and revealed the importance of carbon sources in the growth medium for the production of depolymerase enzymes, as the rate of polymer degradation was affected by the source of carbon. Findings from our CCD model suggests that the autonomous factors like K_2HPO_4, time, KH_2PO_4 and interactive effect of time with PHB were the important variables in the PHB depolymerase enzyme production (Wani et al. 2015). To validate the above study, soil burial method with the significant factors screened from RSM was applied on the PHB film to observe the degradation of PHB polymer film. In the present study, it is observed that the degradation rate of PHB polymer film increased from 85.76% to 94.4% after the PHB film was treated with *A. caviae* Kuk1-(34) sp. in optimized conditions. This remarkable degrading characteristic of the strain was utilized for the soil bioremediation to deal with the solid waste management. Since degradation proceeds at natural environmental conditions of temperature (35-37 ℃), pH 7.0, presence of nutrients in soil and oxygen availability allows faster microbial growth by utilizing PHB film as carbon source. So, soil was found to be the most promising ecosystem for PHB degradation in presence of microbial

activity that is enzymatic degradation is more relevant than composting or chemical treatment for solid waste.

Conclusion

The isolated strain *A. caviae* Kuk1-(34) sp. from sewage waste showed great potential to degrade PHB based biodegradable polymer film almost completely in soil under optimized conditions within limited time period. The growth and the maximum enzyme activity of PHB depolymerase enzyme assay of the isolated strain was observed at the ambient temperature (27°C), indicating that the isolate have the capability to survive well under the natural environmental conditions. SEM and FTIR analysis of the PHB polymer film confirmed the morphological and structural changes due to the biodegradation of the polymer film. The statistical analysis for production optimization recognised the significant factors of the medium for the increased production of PHB depolymerase enzyme that saves time and currency as well. It can be concluded that *A. caviae* Kuk1-(34) sp. isolated from the sewage waste emerges as a potent producer of extracellular PHB depolymerase enzyme, having the potential to act as a bio-catalyst for the biodegradation of PHB-based bioplastics for large scale bio-remediation of soil.

Acknowledgement

The authors are grateful to Integral University for providing the facilities for conducting this work. The authors are very thankful to Deanship of Scientific Research at the Majmaah University for providing the facilities.

Financial Disclosure Statement

Dr. Roohi is grateful to the Department of Science and Technology, India (DST-SERB) for providing financial support under the scheme of Early Career Research Award (Project No. ECR/2017/001001). The funders had no role in study design, data collection and analysis, decision to publish, or preparation of the manuscript.

References

1. Agustin MB, Ahmmad B, Alonzo SMM, Patriana FM. Bioplastic based on starch and cellulose nanocrystals from rice straw. J Reinf Plast Compos. 2014;33: 2205–2213. https://doi.org/10.1177/0731684414558325

2. Altaee N, El-Hiti GA, Fahdil A, Sudesh K, Yousif E. Biodegradation of different formulations of polyhydroxybutyrate films in soil. Springerplus. 2016;5: 762. https://doi.org/10.1186/s40064-016-2480-2

3. Bano K, Kuddus M, Zaheer MR, Roohi. A novel, thermotolerant, extracellular PHB depolymerase producer Paenibacillus alvei PHB28 for bioremediation of biodegradable plastics. Turk J Biochem. 2019;44: 344–353. https://doi.org/10.1515/tjb-2018-0207

4. Bansal S, Soni SK, Harjai K, Chhibber S. Aeromonas punctata derived depolymerase that disrupts the integrity of Klebsiella pneumoniae capsule: Optimization of depolymerase production. J Basic Microbiol. 2014;54: 711–720. https://doi.org/10.1002/jobm.201300356

5. Calil MR. The effect of UV-B irradiation on the biodegradability of poly-β-hydroxybutyrate (PHB) and poly-ε-caprolactone (PCL). Journal of Polymer and the Environment. 2001;9: 109–113. https://doi.org/10.1023/A:1020498710586

6. Chidambarampadmavathy K, Karthikeyan OP, Heimann K. Sustainable bio-plastic production through landfill methane recycling. Renew Sustain Energy Rev. 2017;71: 555–562. https://doi.org/10.1016/j.rser.2016.12.083

7. Elbanna K, Lütke-Eversloh T, Jendrossek D, Luftmann H, Steinbüchel A. Studies on the biodegradability of polythioester copolymers and homopolymers by polyhydroxyalkanoate (PHA)-degrading bacteria and PHA depolymerases. Arch Microbiol. 2004;182: 212–225. https://doi.org/10.1007/s00203-004-0715-z

8. Getachew A, Woldesenbet F. Production of biodegradable plastic by polyhydroxybutyrate (PHB) accumulating bacteria using low cost agricultural waste material. BMC Res Notes. 2016;9: 1–9. https://doi.org/10.1186/s13104-016-2321-y

9. Gouda MK, Swellam AE and Omar SH. Biodegradation of synthetic polyesters (BTA and PCL) with natural flora in soil burial and pure cultures under ambient temperature. Res J Environ Earth Sci. 2012;03: 325-333.

10. Gautam N and Kaur I. Soil burial biodegradation studies of starch grafted polyethylene and identification of Rhizobium meliloti therefrom. J Environ Chem Ecotoxicol. 2013;5(6): 147-158. Doi: 10.5897/JECE09.022.

11. McGenity T, Van Der Meer JR, de Lorenzo V. Handbook of hydrocarbon and lipid microbiology. Springer; 2010.

12. Mergaert J, Webb A, Anderson C, Wouters A, Swings J. Microbial degradation of poly(3-hydroxybutyrate) and poly(3-hydroxybutyrate-co-3-hydroxyvalerate) in soils. Appl Environ Microbiol. 1993;59: 3233-3238. https://doi.org/10.1128/aem.59.10.3233-3238.1993

13. Babu J, Nath SB, Kodali VP. Isolation, screening and extraction of polyhydroxybutyrate (PHB) producing bacteria from sewage sample. Int J Pharm Tech Res. 2014;6: 850-857.

14. Ni J, Wang M. In vitro evaluation of hydroxyapatite reinforced polyhydroxybutyrate composite. Mater Sci Eng C. 2002;20: 101–109. https://doi.org/10.1016/S0928-4931(02)00019-X

15. Ong YT, Ahmad AL, Zein SH, Sudesh K, Tan SH. Poly (3-hydroxybutyrate)-functionalised multi-walled carbon nanotubes/chitosan green nanocomposite membranes and their application in pervaporation. Sep Purif Technol. 2011;76(3): 419-427. https://doi.org/10.1016/j.seppur.2010.11.013

16. Satti SM, Shah Z, Luqman A, Hasan F, Osman M, Shah AA. Biodegradation of Poly(3-

hydroxybutyrate) and Poly(3-hydroxybutyrate-co-3-hydroxyvalerate) by newly isolated Penicillium oxalicum SS2 in soil microcosms and partial characterization of extracellular depolymerase. Curr Microbiol. 2020;77: 1622–1636. https://doi.org/10.1007/s00284-020-01968-7

17. Sayyed RZ, Wani SJ, Alarfaj AA, Syed A, El-Enshasy HA. Production, purification and evaluation of biodegradation potential of PHB depolymerase of Stenotrophomonas sp. RZS7. PLoS One. 2020;15: e0220095. https://doi.org/10.1371/journal.pone.0220095

18. Sayyed RZ, Wani SJ, Alyousef AA, Alqasim A, Syed A, El-Enshasy HA. Purification and kinetics of the PHB depolymerase of Microbacterium paraoxydans RZS6 isolated from a dumping yard. PLoS One. 2019;14: e0212324. https://doi.org/10.1371/journal.pone.0212324

19. Shah AA, Hasan F, Hameed A, Ahmed S. Isolation and characterization of poly(3-hydroxybutyrate-co-3-hydroxyvalerate) degrading bacteria and purification of PHBV depolymerase from newly isolated Bacillus sp. AF3. Int Biodeterior Biodegrad. 2007;60: 109–115. https://doi.org/10.1016/j.ibiod.2007.01.004

20. Sharma B, Jain P. Deciphering the advances in bioaugmentation of plastic wastes. J Clean Prod. 2020;20: 123241. https://doi.org/10.1016/j.jclepro.2020.123241

21. Shivakumar S. Production of Poly (β) hydroxybutyrate (PHB) depolymerase PHAZ from Penicillium expansum by Plackett-Burman design: Partial purification and characterization. Int J Chemtech Res. 2012;4: 430-7.

22. Vogel C, Morita S, Sato H, Noda I, Ozaki Y, Siesler HW. Thermal degradation of poly(3-hydroxybutyrate) and poly(3-hydroxybutyrate- co-3-hydroxyhexanoate) in nitrogen and oxygen studied by thermogravimetric- fourier transform infrared spectroscopy. Appl Spectrosc. 2007;61: 755–764. https://doi.org/10.1366/000370207781393370

23. Wang L, Zhu, W, Wang X, Chen X, Chen GQ, Xu K. Processability modifications of poly(3-hydroxybutyrate) by plasticizing, blending, and stabilizing. J Appl Polym Sci. 2008;107: 166–173. https://doi.org/10.1002/app.27004

24. Wani SJ, Shaikh SS, Sayyed RZ. Medium optimization for PHB depolymerase production by Stenotrophomonas maltophilia using Plackett Burman design & Response Surface Methodology. Int J Sci Eng. 2015;6(10): 818-829.

25. Wani SJ, Shaikh SS, Tabassum B, Thakur R, Gulati A, Sayyed RZ. Stenotrophomonas sp. RZS 7, a novel PHB degrader isolated from plastic contaminated soil in Shahada, Maharashtra, Western India. 3 Biotech. 2016;6: 179. https://doi.org/10.1007/s13205-016-0477-8

26. Wen X, and Lu X. Microbial degradation of Poly(3-Hydroxybutyrate-co-4-Hydroxybutyrate) in soil. J Polym Environ. 2012;20: 381–387. https://doi.org/10.1007/s10924-011-0387-0

27. Wu CS. Characterization and biodegradability of polyester bioplastic-based green renewable composites from agricultural residues. Polym Degrad Stab. 2012;97: 64–71. https://doi.org/10.1016/j.polymdegradstab.2011.10.012

28. Yu J, Plackett D, Chen LXL. Kinetics and mechanism of the monomeric products from abiotic hydrolysis of poly[(R)-3-hydroxybutyrate] under acidic and alkaline conditions. Polym Degrad Stab.

2005;89: 289–299. https://doi.org/10.1016/j.polymdegradstab.2004.12.026

29. Zhou H, Wang Z, Chen S, Liu D, Xia H. Purification and characterization of extracellular poly(β-hydroxybutyrate) depolymerase from Penicillium sp. DS9701-D2. Polym Plast Technol Eng. 2009;48: 58–63. https://doi.org/10.1080/03602550802539627.

Figure legends

Figure 1. Images showing clear zone of hydrolysis of microbial colonies in (A),(B) and Positive colonies showing zone diameter of PHB hydrolysis on PHB agar media [C: CA6-(55), D: CB2-(20), E: Kuk1-(34)]

Figure 2. Phylogenetic tree of *Aeromonas caviae* Kuk1-(34) sp. drawn using UPGMA tree (MEGA X software) with the evolutionary distances showing the relationship of PHB depolymerase producing bacteria with the validity published sequences of related genera.

Figure 3. Degradation of PHB film treated with *Aeromonas caviae* Kuk1-(34) sp. in soil burial as compared with (A) control (B) 15 days (C) 30 days and (D) 45 days

Figure 4. Morphological changes in SEM micrographs of PHB film degraded by *Aeromonas caviae* Kuk1-(34) sp. in soil burial after incubation of (A) Control PHB film without treatment, (B) 15 days (C) 30 days (D) 45 days

Figure 5. FTIR chromatogram of soil buried PHB film after the degradation by *Aeromonas caviae* Kuk1-(34) sp. read between 4000 to 450 cm^{-1}. The functional groups changes were compared with each film after (A) Control PHB film without treatment, (B) 15 days (C) 30 days (D) 45 days

Figure 6. Pareto chart of the significant factor in the (A) PlackettBurman design (B) Central composite design for the enzyme production (C) Surface plot of enzyme activity of the bacterial strain *Aeromonas caviae* Kuk1-(34) sp. (D) Contour plot showing the interaction effect of temperature and time on PHB depolymerase enzyme production by *Aeromonas caviae* Kuk1-(34) sp.

Figure 7. Soil burial method for the degradation of PHB film as compared with (A) control after treatment with the *Aeromonas caviae* Kuk1-(34) sp. for (B) 15 days (C) 30 days and (D) 45 days.

Figure Click here to access/download;Figure;Figures-Aeromonas caviae (20.08.21).doc

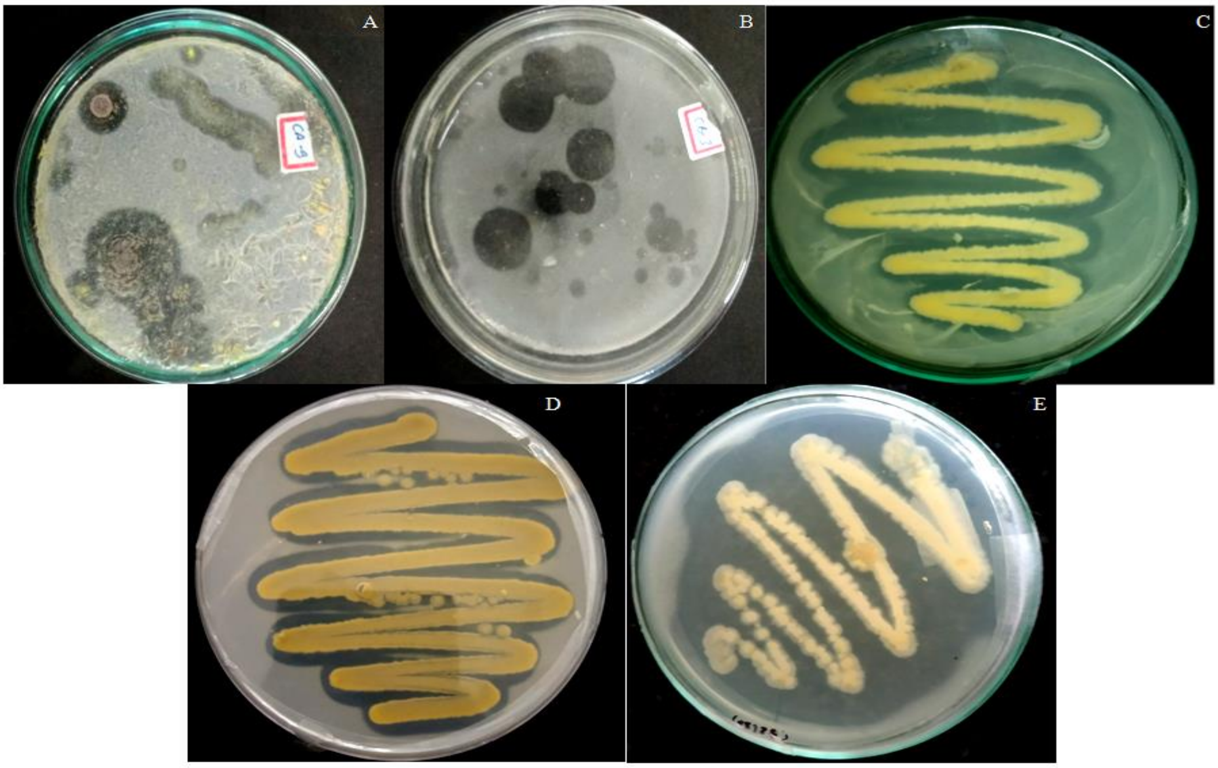

Figure 1

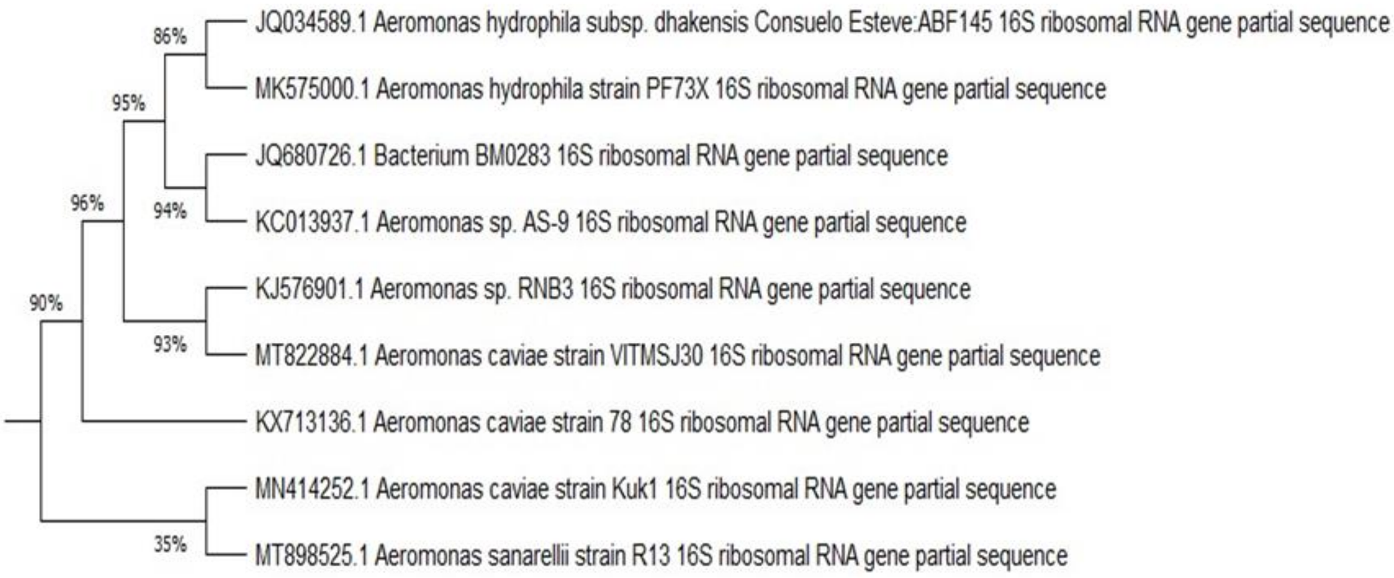

JQ034589.1 Aeromonas hydrophila subsp. dhakensis Consuelo Esteve:ABF145 16S ribosomal RNA gene partial sequence
MK575000.1 Aeromonas hydrophila strain PF73X 16S ribosomal RNA gene partial sequence
JQ680726.1 Bacterium BM0283 16S ribosomal RNA gene partial sequence
KC013937.1 Aeromonas sp. AS-9 16S ribosomal RNA gene partial sequence
KJ576901.1 Aeromonas sp. RNB3 16S ribosomal RNA gene partial sequence
MT822884.1 Aeromonas caviae strain VITMSJ30 16S ribosomal RNA gene partial sequence
KX713136.1 Aeromonas caviae strain 78 16S ribosomal RNA gene partial sequence
MN414252.1 Aeromonas caviae strain Kuk1 16S ribosomal RNA gene partial sequence
MT898525.1 Aeromonas sanarellii strain R13 16S ribosomal RNA gene partial sequence
86%
95%
94%
96%
90%
93%
35%

Figure 2

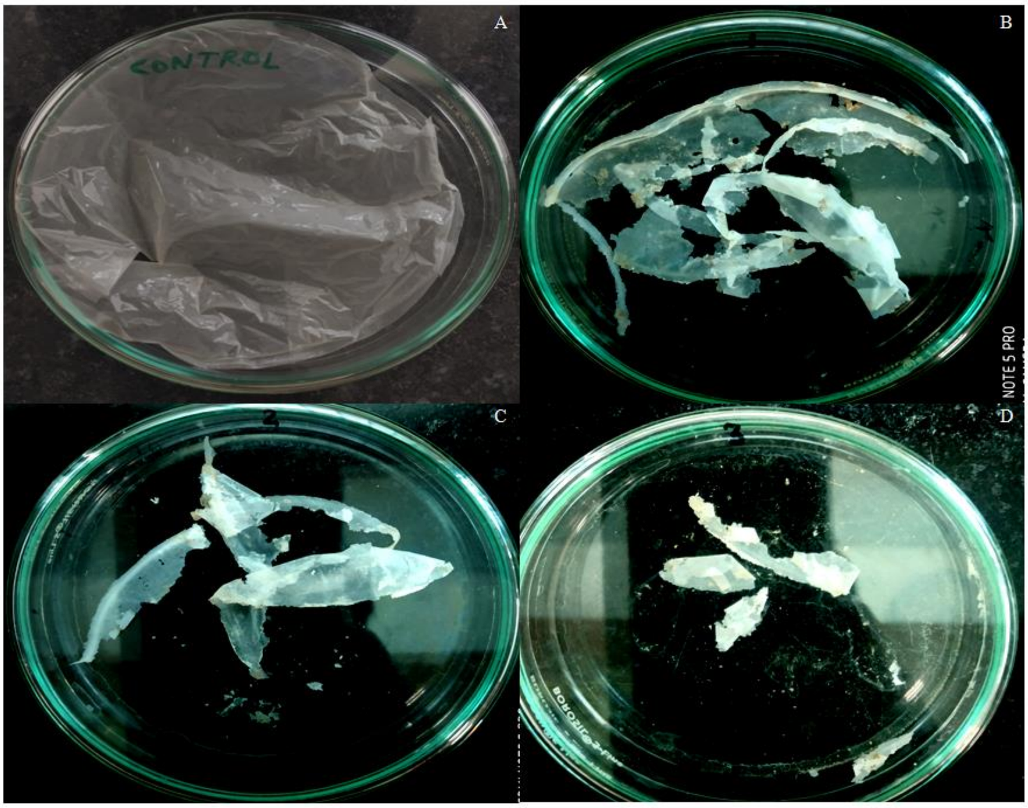

Figure 3

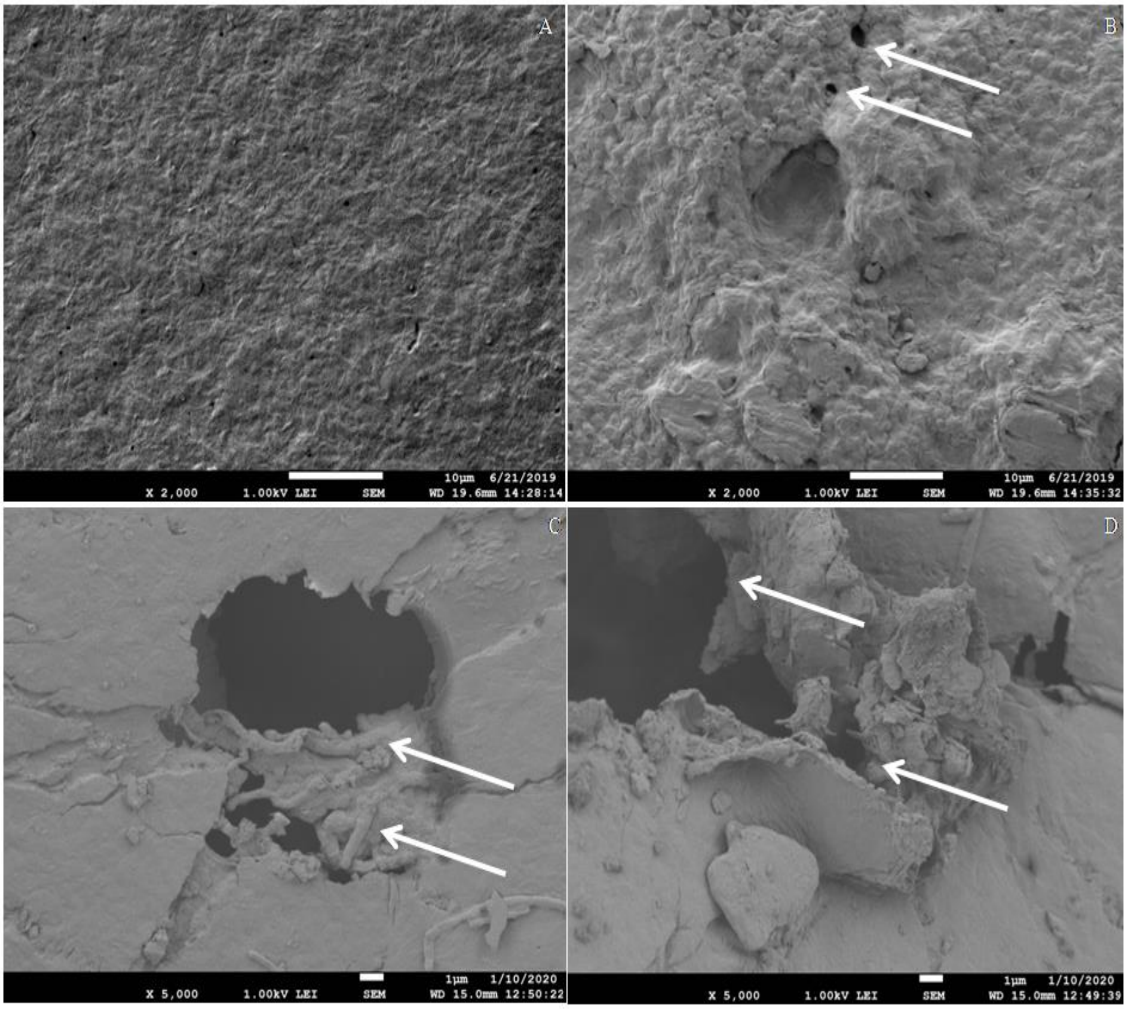
A
10μm 6/21/2019
X 2,000 1.00kV LEI SEM WD 19.6mm 14:28:14
B
10μm 6/21/2019
X 2,000 1.00kV LEI SEM WD 19.6mm 14:35:32
C
1μm 1/10/2020
X 5,000 1.00kV LEI SEM WD 15.0mm 12:50:22
D
1μm 1/10/2020
X 5,000 1.00kV LEI SEM WD 15.0mm 12:49:39

Figure 4

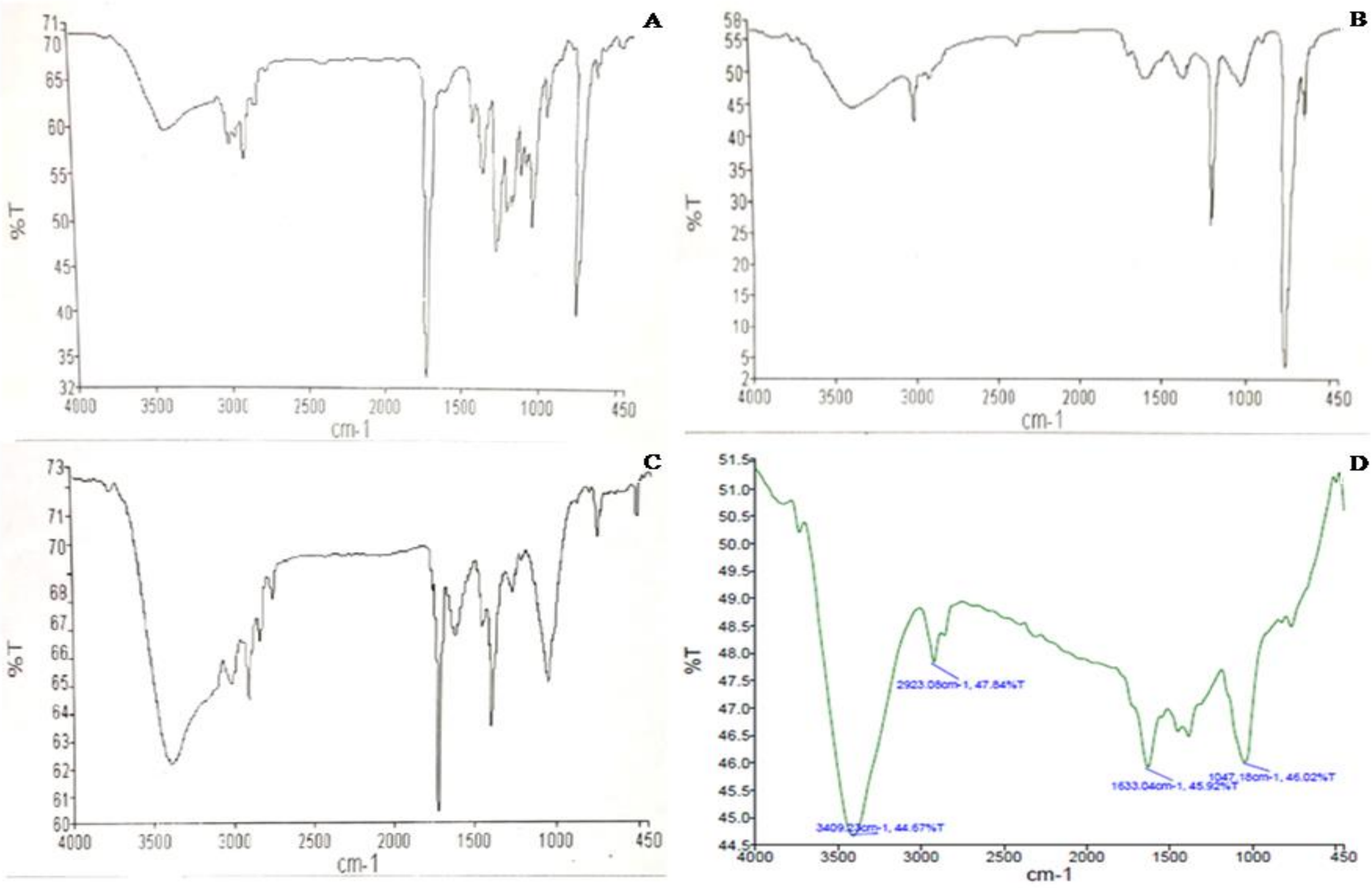
A
B
C
D
%T
cm-1
2923.08cm-1, 47.84%T
3409.23cm-1, 44.67%T
1633.04cm-1, 45.92%T
1047.18cm-1, 46.02%T

Figure 5

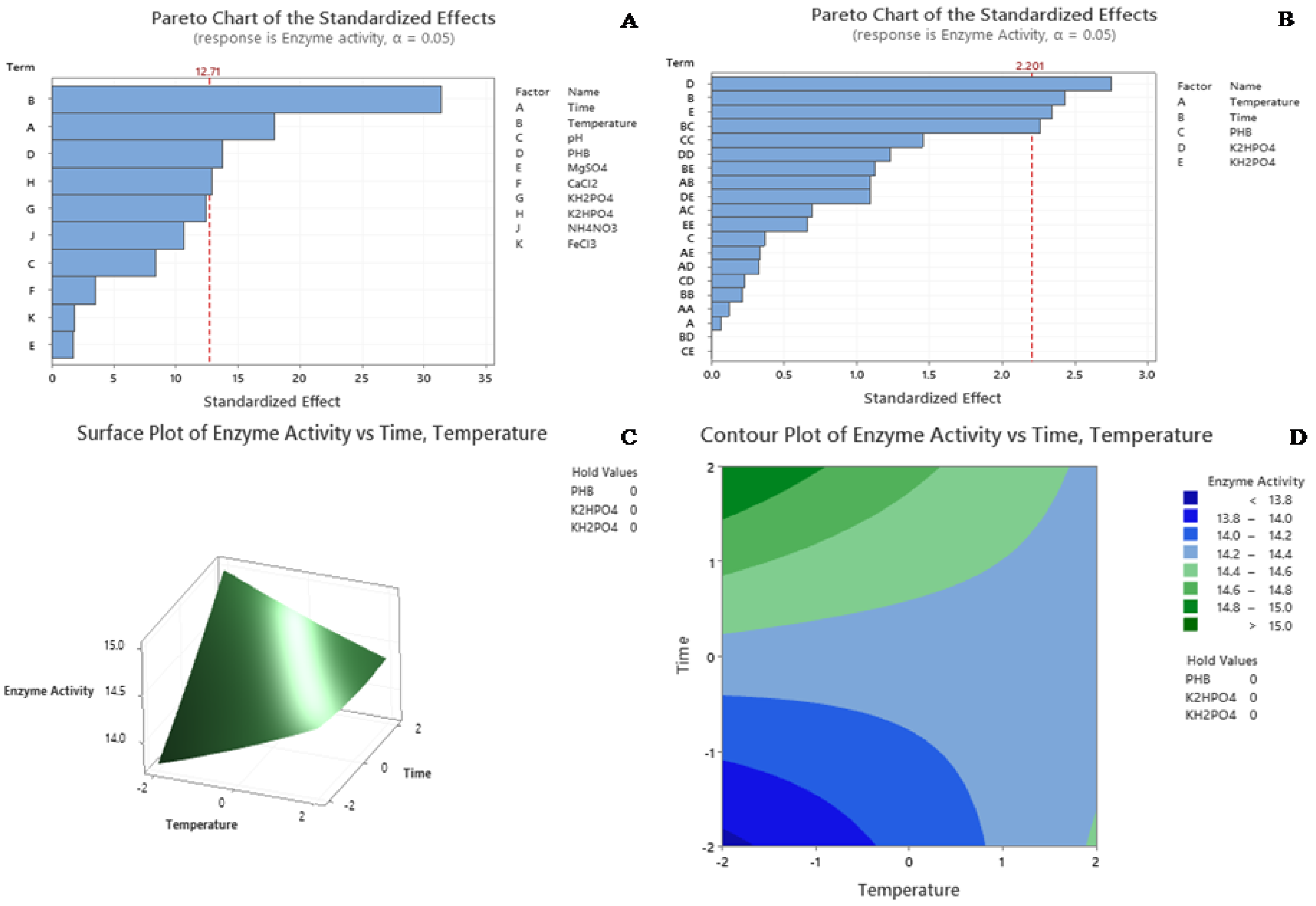

Pareto Chart of the Standardized Effects
(response is Enzyme activity, α = 0.05)
A
Term
12.71
B
A
D
H
G
J
C
F
K
E
Factor Name
A Time
B Temperature
C pH
D PHB
E MgSO4
F CaCl2
G KH2PO4
H K2HPO4
J NH4NO3
K FeCl3
0 5 10 15 20 25 30 35
Standardized Effect
Pareto Chart of the Standardized Effects
(response is Enzyme Activity, α = 0.05)
B
Term
2.201
D
B
E
BC
CC
DD
BE
AB
DE
AC
EE
C
AE
AD
CD
BB
AA
A
BD
CE
Factor Name
A Temperature
B Time
C PHB
D K2HPO4
E KH2PO4
0.0 0.5 1.0 1.5 2.0 2.5 3.0
Standardized Effect
Surface Plot of Enzyme Activity vs Time, Temperature
C
Hold Values
PHB 0
K2HPO4 0
KH2PO4 0
Enzyme Activity
15.0
14.5
14.0
-2 0 2
Temperature
2 0 -2
Time
Contour Plot of Enzyme Activity vs Time, Temperature
D
Time
2 1 0 -1 -2
-2 -1 0 1 2
Temperature
Enzyme Activity
< 13.8
13.8 – 14.0
14.0 – 14.2
14.2 – 14.4
14.4 – 14.6
14.6 – 14.8
14.8 – 15.0
> 15.0
Hold Values
PHB 0
K2HPO4 0
KH2PO4 0

Figure 6

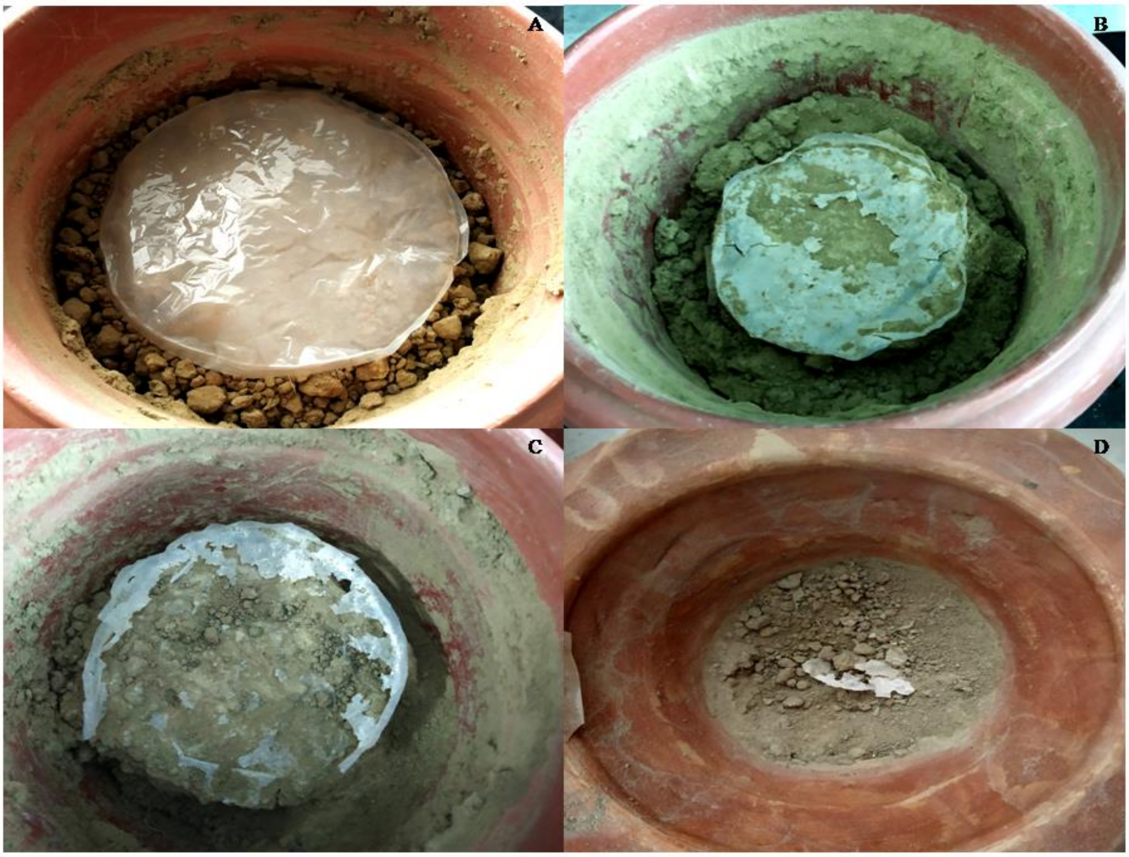

Figure 7

**Table 1 (A) Component design of PlackettBurman for PHB depolymerase production with the bacterial strain *Aeromonas caviae* Kuk1-(34) sp.**				
**Variable**	**Symbol**	**(+1) High value**	**(0) Central value**	**(-1) Low Value**
Time (days)	A	7	5	3
Temp (ºC)	B	47º	37º	27º
pH	C	9	7	5
PHB (%)	D	0.2	0.15	0.1
$MgSO_4$ (g/L)	E	0.25	0.20	0.15
$CaCl_2$ (g/L)	F	0.005	0.003	0.001
KH_2PO_4(g/L)	G	1.3	1	0.7
K_2HPO_4 (g/L)	H	1.3	1	0.7
NH_4NO_3 (g/L)	I	1.3	1	0.7
$FeCl_3$ (g/L)	J	0.07	0.05	0.03

**Table 1 (B) Component design of CCD for PHB depolymerase production with the bacterial strain *Aeromonas caviae* Kuk1-(34) sp.**						
**Variable**	**Symbol**	**(+2) value**	**(+1) value**	**(0)**	**(-1) Value**	**(-2) Value**
Time (days)	A	9	7	5	3	1
Temp(ºC)	B	57	47	37	27	17
PHB (%)	D	0.25	0.2	0.15	0.1	0.05
KH_2PO_4(g/L)	G	1.6	1.3	1	0.7	0.4
K_2HPO_4(g/L)	H	1.6	1.3	1	0.7	0.4

Table 2. Soil samples taken from different locations of Lucknow, UP, India

Sampling site	Latitude/Longitude	Total Isolates	Positive Isolates
Kukrail	26.91 N/ 80.98 E	73	15
Gomti Nagar	26.84 N/ 81.00 E	31	04
IIM road	26.80 N/ 80.76 E	16	02
Molviganj	26.85 N/ 80.92 E	07	01

Table 3 (A). Enzyme activity and CFU count of positive bacterial isolates							
**S. No**	**Positive isolates**	**Colony size in mm (a)**	**Zone of hydrolysis in mm (b)**	**Zone diameter in mm (b/a)**	**Enzyme activity (U/mL/min)**	**Decrease in turbidity measured at OD_{650}**	**CFU/mL**
1	Control	NA	NA	NA	NA	0.823	NA
2	CA6-(55)	4	10	2.5	1.55	0.379	1.29×10^9
3	CB2-(20)	4	24	6	1.71	0.404	4.80×10^9
4	Kuk1-(34)	3	34	11.3	2.06	0.320	4.10×10^9

Table 3 (B). Morphological, biochemical and physiological characteristics of the bacterial strain Kuk1-(34)	
**Characteristics**	**Bacterial strain Kuk1-(34)**
**Morphological tests**	
Grams staining	Negative
Pigmentation	Yellow
Form	Irregular
Elevation	Umbonate
Cell shape	Rod
Margin	Undulate
**Biochemical tests**	
Cellulose	+
Casein	+
Indole	-
Methyl red	-
Voges-Proskauer	-
Citrate	+
Hydrogen sulphide	+
Catalase	+
**Physiological tests**	
Growth at temperature	17ºC-37ºC
Growth at NaCl (%)	0.5-5
Growth at pH	6.0-8.0

Table 4. 16S rRNA identification of positive isolates

S. No	Submitted microbial strain	Identity	Accession Number	Similarity Index %
1.	CA6-(55)	*Enterobacter cloacae* CA655	MN088848	97.88
2.	CB2-(20)	*Stenotrophomonas* sp.CB220	MN736124	96.60
3.	Kuk1-(34)	*Aeromonas caviae*Kuk1sp.	MN414252	99.43

Table 5. Weight loss degradability of PHB film after different incubation time

Bacterial species	Weight of PHB film (gm)	Weight of pre-treated soil buried PHB film (gm)			Weight loss degradability
		15 days	30 days	45 days	After 45 days (%)
Control	0.0240	0.0240	0.0240	0.0240	0.00%
CA6-(55)	0.0250	0.0224	0.0190	0.0145	42.00%
CB2-(20)	0.0240	0.0229	0.0202	0.0170	29.16%
Kuk1-(34)	0.0260	0.0232	0.0187	0.0037	85.76%

Table 6 (A). Placket Burman experimental design for PHB depolymerase production with the bacterial strain *Aeromonas caviae* Kuk1-(34) sp.

Run Order	(A)	(B)	(C)	(D)	(E)	(F)	(G)	(H)	(I)	(J)	Enzyme activity (U/ml)
1	-1	1	-1	-1	-1	1	1	1	-1	1	3.467
2	1	1	-1	1	1	-1	1	-1	-1	-1	0.967
3	-1	-1	-1	1	1	1	-1	1	1	-1	9.8
4	-1	1	1	1	-1	1	1	-1	1	-1	0.701
5	1	1	1	-1	1	1	-1	1	-1	-1	1.048
6	1	-1	-1	-1	1	1	1	-1	1	1	1.88
7	1	1	-1	1	-1	-1	-1	1	1	1	3.79
8	-1	-1	1	1	1	-1	1	1	-1	1	9.25
9	1	-1	1	-1	-1	-1	1	1	1	-1	2.9
10	1	-1	1	1	-1	1	-1	-1	-1	1	6.153
11	-1	1	1	-1	1	-1	-1	-1	1	1	0.887
12	-1	-1	-1	-1	-1	-1	-1	-1	-1	-1	8.38

Table 6(B). ANOVA analysis for PlackettBurman design for PHB depolymerase enzyme production

Source	DF	Adj SS	Adj MS	F-Value	*p*-Value
Model	10	129.354	12.9354	202.74	0.055
Linear	10	129.354	12.9354	202.74	0.055
Time (Days)	1	20.664	20.6640	323.88	0.035
Temperature (ºC)	1	63.035	63.0346	987.97	0.020
pH	1	4.496	4.4958	70.46	0.075
PHB (%)	1	12.199	12.1988	191.20	0.046
$MgSO_4$ (g/L)	1	0.203	0.2025	3.17	0.326
$CaCl_2$(g/L)	1	0.814	0.8138	12.76	0.174
KH_2PO_4(g/L)	1	9.888	9.8881	154.98	0.051
K_2HPO_4(g/L)	1	10.616	10.6164	166.40	0.049
NH_4NO_3(g/L)	1	7.218	7.2184	113.14	0.060
$FeCl_3$(g/L)	1	0.222	0.2217	3.47	0.313
Error	1	0.064	0.0638		
Total	11	129.418			

Table 7 (A). Central Composite Design for PHB depolymerase production with the bacterial strain *Aeromonas caviae* Kuk1-(34) sp.

Run Order	A	B	D	G	H	Enzyme activity (U/mL)	
						Predicted value	Experimental value
1	0	0	0	2	0	13.87346	13.87
2	-1	1	-1	-1	-1	14.14433	14.09
3	0	-2	0	0	0	14.32713	14.71
4	0	0	0	0	0	14.30838	14.11
5	0	2	0	0	0	14.34463	13.98
6	1	1	1	-1	-1	14.29292	14.38
7	-1	-1	1	1	1	14.23675	14.17
8	1	1	-1	-1	1	14.60967	14.80
9	-2	0	0	0	0	14.05696	14.29
10	0	0	0	0	0	14.30838	14.1
11	1	1	-1	1	-1	14.11608	14.18
12	0	0	0	0	0	14.30838	14.47
13	1	-1	-1	-1	-1	14.60925	14.48
14	-1	-1	-1	1	-1	13.40492	13.17
15	0	0	0	0	-2	14.24346	14.53
16	-1	1	1	1	-1	13.99858	13.98
17	-1	-1	-1	-1	1	14.2325	14.13
18	2	0	0	0	0	14.65479	14.44
19	-1	-1	1	-1	-1	14.29175	14.08
20	-1	1	-1	1	1	13.95933	14.05
21	0	0	0	0	0	14.30838	14.41
22	0	0	0	0	0	14.30838	14.53
23	0	0	0	0	2	14.91829	14.65
24	0	0	0	-2	0	14.44829	14.47
25	1	-1	1	1	-1	14.0125	13.91
26	1	-1	1	-1	1	14.43308	14.47
27	1	1	1	1	1	14.37792	14.61
28	0	0	0	0	0	14.30838	14.2
29	1	-1	-1	1	1	14.96425	14.98
30	0	0	-2	0	0	13.93996	14.008
31	-1	1	1	-1	1	14.75617	14.87
32	0	0	2	0	0	14.02979	13.98

Table 7(B). ANOVA analysis of CCD for PHB depolymerase enzyme production

Source	DF	Adj SS	Adj MS	F-Value	*p*-Value
Model	20	2.99525	0.149763	1.66	0.195
Linear	5	1.72742	0.345484	3.82	0.030
Temperature (ºC)	1	0.00046	0.000459	0.01	0.944
Time (Days)	1	0.53611	0.536107	5.93	0.033
PHB (%)	1	0.01211	0.012105	0.13	0.721
K_2HPO_4 (g/L)	1	0.68310	0.683100	7.56	0.019
KH_2PO_4 (g/L)	1	0.49565	0.495650	5.49	0.039
Square	5	0.40545	0.081090	0.90	0.516
Temperature* Temperature	1	0.00139	0.001386	0.02	0.904
Time*Time	1	0.00414	0.004136	0.05	0.834
PHB*PHB	1	0.19186	0.191862	2.12	0.173
K_2HPO_4*K_2HPO_4	1	0.13614	0.136136	1.51	0.245
KH_2PO_4*KH_2PO_4	1	0.03989	0.039886	0.44	0.520
2-Way Interaction	10	0.86238	0.086238	0.95	0.525
Temperature*Time	1	0.10808	0.108077	1.20	0.297
Temperature*PHB	1	0.04337	0.043368	0.48	0.503
Temperature*K_2HPO_4	1	0.00985	0.009851	0.11	0.747
Temperature*KH_2PO_4	1	0.01015	0.010151	0.11	0.744
Time*PHB	1	0.46410	0.464102	5.14	0.045
Time*K_2HPO_4	1	0.00001	0.000005	0.00	0.994
Time*KH_2PO_4	1	0.11408	0.114075	1.26	0.285
PHB*K_2HPO_4	1	0.00501	0.005006	0.06	0.818
PHB*KH_2PO_4	1	0.00000	0.000001	0.00	0.998
KH_2PO_4* KH_2PO_4	1	0.10775	0.107748	1.19	0.298
Error	11	0.99380	0.090345		
Lack-of-Fit	6	0.81247	0.135412	3.73	0.085
Pure Error	5	0.18133	0.036265		
Total	31	3.98905			

Table 8. Weight loss degradability of PHB film after different incubation time

Bacterial species	Weight of PHB film (g)	Weight of pre-treated soil buried PHB film (g)			Weight loss degradability (%) after 45 days
		15 days	30 days	45 days	
Control	0.0250	0.0250	0.0250	0.0250	0.00%
Aeromonas caviae Kuk1-(34) sp.	0.0250	0.0198	0.0089	0.0014	94.4%
