# Supplementary material for: Isolation and optimization of extracellular PHB depolymerase producer Aeromonas caviae Kuk1-(34) for sustainable solid waste management of biodegradable polymers
Source: PLoS One. 2022 Apr 14;17(4):e0264207. doi: 10.1371/journal.pone.0264207 (PMC9009665; doi:10.1371/journal.pone.0264207)
Supplement: Attachment — Submitted filename: Reviewers Report PONE-D-21-27980.docx [file pone.0264207.s002.docx]

**REVIEWER REPORT OF MS No. PONE-D-21-27980**

1. Authors did an excellent job of explaining the concept clearly and accurately.
2. Author should be congratulated for the novel endeavour, however language throughout the MS has to be improved, typographical errors, grammatical errors and syntax errors should be removed before submission.
3. Uniformity should be maintained, while writing the author's name.
4. There is a variation of statement between line no. 43 & 52. Author should check that properly.
5. In line no. 89, 'soil waste' should be replaced by 'sewage waste soil'.
6. In line no. 93, author should only mention the abbreviated form of 'PHB', as the full form is already mentioned in the introduction section.
7. Recast line no. 140.
8. The sub-section 2.11. should be deleted as this protocol is already mentioned in the sub-section 2.7. Author should properly check and modify that.
9. Sub-section 3.1. should be merged with 3.2. and in line no. 198 'soil sample' should be replaced by 'sewage waste soil sample'.
10. In line no. 201, before screening how did they get confirmed that all the 22 isolates were PHB degraders? Author should rearrange the result.
11. In Fig. 1, how the author measure zone of diameter by continuous streaking?
12. In line no. 141, it is already mentioned that 0.0245 g PHB film was used for soil burial, then why term 'different' is used in line no. 222.
13. If only *A. caviae* Kukl-(34) strain was examined for PHB film degradation, then why did author stated that different strains were examined in line no. 223?
14. Author should properly explain the requirement of section 4. This section includes the results, which were already stated in the individual section.
15. Result pertaining to percentage of PHB degradation should be mentioned in sub-section 3.5. instead of section 4, line no. 322.
16. The total weight of PHB degraded after pre-treatment with *A. caviae* Kukl-(34) varied in line no. 43, 52, 322 & 349. Author should check it properly.
17. In line no. 352, insert space between 'Bano' and 'et al'.
18. All the tables should be arranged as per the sequence of result throughout the MS. For eg. Table 2 should be captioned as Table 1 as it is the first table in the result section.
19. Reports are available (Pati et al., 2020, https://doi.org/10.1007/s00284-020-01922-7) for PHB degradation within 7-21 days by composting method. Thus, author should compare degradation of PHB in laboratory as well as environmental condition.
20. Throughout the MS, term 'the' is used repeatedly. Author should look into that and rectify wherever necessary.

*The MS is accepted for publication; however, minor modification is required as per suggestion.*
